# Purinergic signaling during Marek’s disease in chickens

**DOI:** 10.1038/s41598-023-29210-x

**Published:** 2023-02-04

**Authors:** Haji Akbar, Julia J. Fasick, Nagendraprabhu Ponnuraj, Keith W. Jarosinski

**Affiliations:** Department of Pathobiology, College of Veterinary Medicine, University of Illinois at Urbana-Champaign, Urbana, IL USA

**Keywords:** Virology, Herpes virus, Viral host response

## Abstract

Purinergic receptors (PRs) have been reported as potential therapeutic targets for many viral infections including herpesviruses, which urges the investigation into their role in Marek’s disease (MD), a herpesvirus induced cancer in chickens that is an important pathogen for the poultry industry. MD is caused by MD virus (MDV) that has a similar viral life cycle as human varicella zoster virus in that it is shed from infected epithelial skin cells and enters the host through the respiratory route. In this report, PR responses during natural MDV infection and disease progression was examined in MD-resistant white Leghorns (WL) and MD-susceptible Pure Columbian (PC) chickens during natural infection. Whole lung lavage cells (WLLC) and liver tissue samples were collected from chickens infected but showing no clinical signs of MD (Infected) or presenting with clinical disease (Diseased). RNA was extracted followed by RT-qPCR analysis with gene specific primers against members of the P1, P2X, and P2Y PR families. Differential expression (*p* < 0.05) was observed in breed and disease conditions. Some PRs showed tissue specific expression (P1A1, P2X1, and P2X6 in WLLC) whereas others responded to MDV infection only in MD-susceptible (PC) chickens (P1A2A, P2X1, P2X5, P2X7). P2Y PRs had differential expression in both chicken lines in response to MDV infection and MD progression. This study is the first to our knowledge to examine PR responses during MDV infection and disease progression. These results suggest PR signaling may an important area of research for MDV replication and MD.

## Introduction

Marek’s disease (MD) is a highly contagious disease of chickens, caused by Marek’s disease herpesvirus (MDV) or *Gallid alphaherpesvirus* 2^1^. MD has been partially controlled since 1969 through widespread use of vaccines that reduce lymphoma and viral replication but do not prevent viral shedding and spread within a flock^2^. The estimated economic impact of the prevention measures of MDV on the global poultry industry costs more than a billion US$ annually^3^. MDV infection is initiated through the respiratory route either by direct bird to bird contact or indirectly by inhalation of dust and dander from birds shedding infected virus^4^. The main target cells for MDV infection are associated with the host immune system (*i.e.*, B and T lymphocytes, and macrophages) that results in notable release of different cytokines, especially during the primary and reactivation phases of infection^5^. Differences in genetic resistance to MD was first reported over 80 years ago^6^ resulting in selection and breeding of chickens for relative resistance and susceptibility to MD over time^7^.

In addition to acting as an energy source and the building blocks of nucleic acids, ATP, and its metabolites (ADP, cAMP, adenosine), have long been accepted as extracellular signaling molecules (eATP) in both physiological and pathological conditions. Infected or damaged cells release eATP into the extracellular environment of the damaged cells as a “find me signal”^8,9^. This eATP response counters bacterial, fungal, or viral infections by acting as autocrine and paracrine molecules on the cells responsible for its release and neighboring cells. After release into the extracellular environment, these purines (ATP and its metabolites) bind to a distinct class of membrane bound receptors collectively known as the purinergic receptors (PRs)^8^. These receptors are ubiquitously expressed in cells, including the target cells of MDV, and are key players of a wide array of biological processes, including neuromodulation, inflammation, endothelial-mediated vasodilatation, cell migration, wound healing, cell proliferation, differentiation, and apoptosis^10^. This vast PR family has been divided into P1 and P2 based on their binding ability to either adenosine or ATP/UTP, respectively. P1 receptors are comprised of four subfamilies (A1, A2A, A2B, and A3), while P2 receptors are divided into two subfamilies, P2X and P2Y, which are further divided into P2X (1–7) channels, activated by ATP, and G protein-coupled metabotropic P2Y (1–14) receptors^11–13^. Most of these receptors have been identified in chickens, at least at the genomic level. In addition to the above classifications and subdivisions, purinergic responses can be either early (short) or late (long) acting based on the infection and physiological condition of host^14^.

The activation of PRs can be either pro- or anti-infection^15^. Chen et al*.*^16^, recently demonstrated that the P2Y2 receptor increases replication of human cytomegalovirus (HCMV), while P2X5 inhibited HCMV replication. Similar differential response patterns have been seen where P2X7R activation enhances immune killing of tumor cells^17^, while A2BR signaling contributes to immunosuppression in tumors^18^. Zhang and colleagues^19^ demonstrated that P2Y13 is a potential antiviral target to restrict the replication of different types of viruses including Newcastle disease virus (NDV) and herpes simplex virus 1 (HSV1). The differential expression of PRs has previously been observed in other pathological conditions^20,21^. All this information, in combination with the clinical manifestation of MD and the functional changes induced in target cells (B and T cells) suggests potential roles for PRs (P1 and P2) in MD pathogenesis. Only a few studies have reported on the expression of PRs in response to herpesvirus infection^11,16,22^, and thus far no papers have been published on MDV and PRs.

MDV is a global economic threat to the poultry industry, but we still lack considerable understanding of pathophysiological mechanisms involved in MD. Current vaccines reduce replication and lymphoma development, but do not induce sterilizing protection^23^. Keeping in view that in-depth understanding of the parameters and signaling pathways involved in disease development are key points in developing better antiviral therapies^24^. The recent finding that P2Y2 and P2X5 are involved in controlling HCMV replication^16^ suggests PRs may play a role other herpesvirus infections, including MD. We hypothesize that: (1) PR expression is significantly changed during MDV infection in chickens; (2) the genetic background of the host chicken plays an important role in PR regulation, and (3) the regulation of these receptors is linked to disease progression and severity. To address this research gap, we used the natural MDV-chicken model to measure PRs mRNA responses during herpesvirus replication and disease progression. The respiratory tract is the natural route of entry for MDV where the virus initially infects pulmonary macrophages and B cells^25^, therefore whole lung lavage cells (WLLC) samples were used in our study, in addition to liver samples due to their importance as a major metabolic organ. Our results reveal differential PRs responses to MDV infection and disease progression when comparing both tissues and breed of chicken.

## Materials and methods

### Cell culture

All cells were maintained in humified atmosphere of 5% CO_2_ at 38 °C. Chick embryo cells (CECs) were prepared as reported earlier^26^. DF-1-Cre cells and have been previously described^27^ and were maintained in LM-based media with Zeocin^28^.

### Generation of rMDV (v2001)

A dually fluorescent recombinant (r)MDV was generated using two fluorescent markers previously shown to have no effect on MDV replication^29,30^. Briefly, the coding sequence of the eGFP gene was inserted in frame at the C-terminus of the MDV UL47 gene by two-step Red-mediated mutagenesis^31^ in the previously described rMDV termed rRLORF4mRFP^28^ bacterial artificial chromosome (BAC) clone exactly as previously described^30^. The BAC clone was named 2001. All BAC clones were confirmed by restriction fragment length polymorphism analysis, analytic PCR, and DNA sequencing (Data not shown). Recombinant viruses (designated with a “v”) were reconstituted by transfecting DF-1-Cre cells using Lipofectamine reagent (Thermo Fisher) and then passaged directly into CECs and used at ≤ 5 passages for in vitro and in vivo studies.

### Immunofluorescence assays (IFAs)

CECs were infected with reconstituted viruses in 6-well tissue culture plates at 100 plaque-forming units (PFU) per well. At 5 days post-infection (dpi), cells were fixed with PFA buffer (2% paraformaldehyde, 0.1% Triton X-100) for 15 min and then washed twice with PBS. The plaques were fluorescent red and green but for double confirmation the fixed cells also were stained with anti-MDV chicken sera plus goat anti-chicken IgY-Alexa Fluor 488 secondary antibody (Molecular Probes, Eugene, OR, USA). The virus plaques were observed using an EVOS FL Cell Imaging System (Thermo Fisher Scientific, Waltham, MA, USA) and compiled using Adobe Photoshop version 21.0.1.

### Plaque size assays (PSA)

Plaque areas were measured in CECs exactly as previously described^32^ using anti-MDV chicken sera and goat anti-chicken IgY-Alexa Fluor 488 secondary antibody (Molecular Probes, Eugene, OR). Digital images of 36 individual plaques were collected using Nikon Eclipse-Ti-E inverted fluorescent microscope and plaque areas were measured using ImageJ^33^ version 1.41o software. Whisker plots were generated using Microsoft Excel 365 and significant differences were determined using IBM SPSS Statistics Version 28 software Package (https://www.ibm.com/analytics/spss-statistics-software).

### Testing virulence of v2001 in chickens

Commercial SPF White Leghorn (WL) chickens were obtained from Hy-Line International (Dallas Center, Iowa) and were from MD-vaccinated parents (maternal antibody positive. Eighteen-day old chicks were inoculated intraabdominally with 1000 plaque forming units (PFU) of vRLORF4mRFP, vUL47eGFP, or v2001 in separate rooms for each group. An additional five chickens were left uninfected to act as contact controls to confirm each virus was able to transmit to uninfected chickens. Chickens were evaluated daily for symptoms of MD and euthanized when birds showed clinical signs of MD (*e.g.*, lethargy, depression, paralysis, etc.) plus examined for gross MD lesions. Chickens positive for MD included birds succumbing to disease prior to the experimental termination date and birds positive for MD-related lesions at termination of the experiment. Fisher’s exact tests were used to determine statistical differences between groups of chickens for MD incidence at a significance level of *p* < 0.05.

### Experimental approach for measuring PR responses

**Figure 1:**
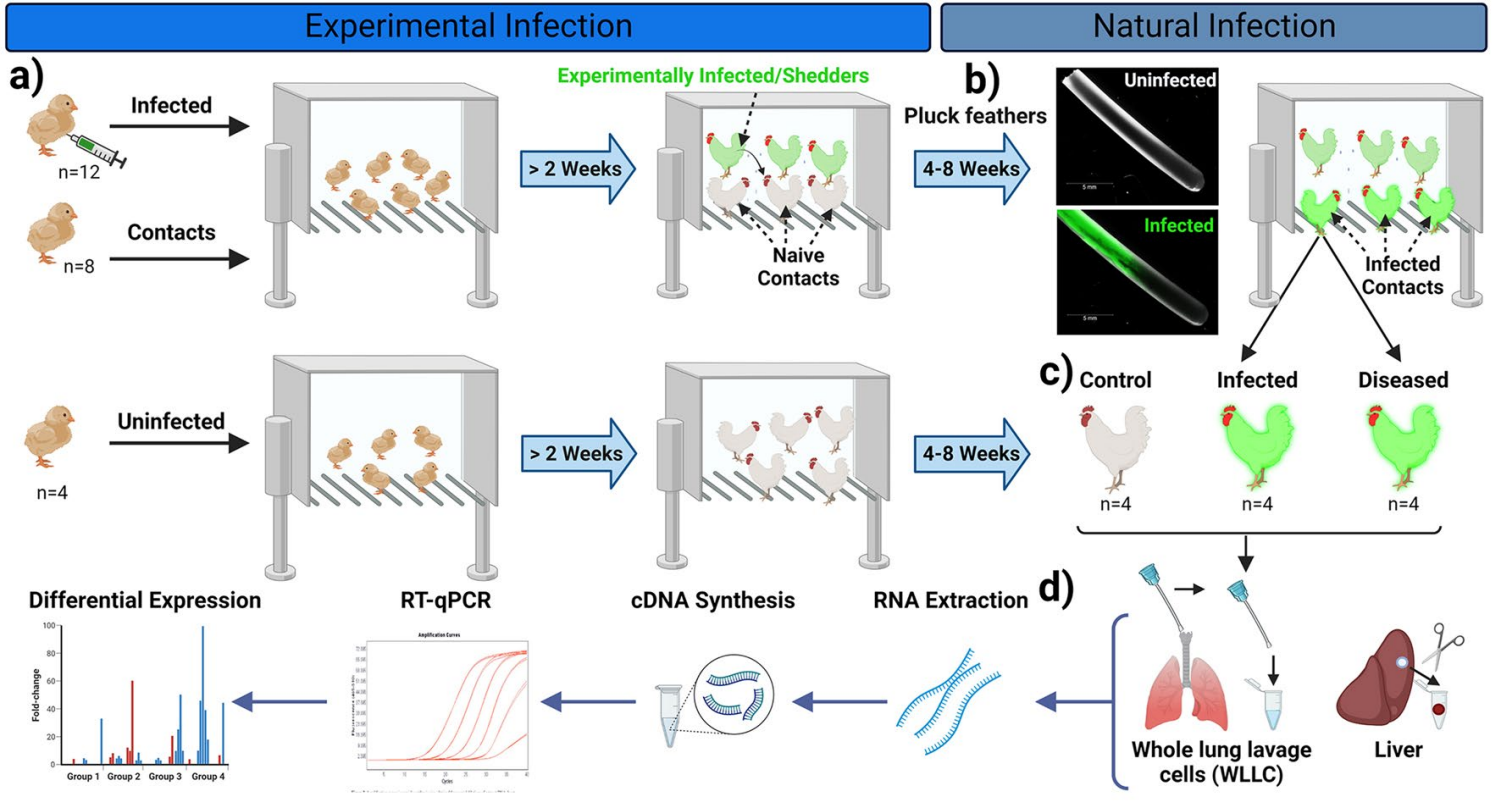
Experimental approach. (**a**) Day old PC (n = 12) and WL (n = 12) chicks were experimentally infected with v2001 by intraabdominal inoculation and housed with naïve contact chicks (n = 8/chicken line) considered “experimental infection”. (**b**) After 2 weeks, MDV is shed into the environment, and naïve contact birds are infected considered “natural infection”. (**c**) Both “Infected”, based on fluorescent feathers and “Diseased”, based on clinical disease, were used for collection of whole lung lavage cell (WLLC) and liver sample collection. (**d**) RNA was extracted, cDNA synthesized, and RT-qPCR was performed to determine differential mRNA expression of PRs.

Day old chickens from two different chicken lines [Pure Columbian (PC); Susceptible (n = 60) and WL; Resistant (n = 60)] were purchased from the UIUC Poultry Research Farm. The major histocompatibility complex (MHC) haplotype for PC birds is not defined, but suggested to be B6-like, while WL are B2/B12^34^. Both chicken lines were housed in separate rooms. MDV is strictly cell-associated during cell culture propagation; therefore, we utilized our established experimental and natural infection model^35^ with some minor modifications. Briefly, three-days old chicks (n = 12 per chicken line) were “experimentally infected” by intraabdominal inoculation of 2,000 PFU of v2001 that mimics disease progression but bypasses the natural respiratory route. For “natural infection,” age-matched, naïve contact (n = 8 per chicken line) chickens from the same breed were also housed with experimentally infected chickens. Approximately 2 weeks after experimental infection, chickens shed infectious virus that can then infect naïve contact chickens, constituting natural infection. Another two sets (one per chicken line) of age-matched uninfected chickens (n = 4) were kept in two separate rooms to be used as uninfected control groups (Fig. 1).

All the birds were screened weekly for infection by visualizing feathers for fluorescently tagged MDV as described previously^36,37^. All chickens were monitored for clinical disease signs such that we had samples at least one week prior to disease manifestation; however, typically symptoms progress over several days^38^. All MDV positive contact birds (naturally infected) were euthanized at different stages of disease progression based on clinical manifestations and were assigned to two groups (n = 4/group/line): Infected (no disease) or Diseased (Tumorigenic). Control birds were euthanized at end of experiment; therefore, a total of three groups were used for analysis; Uninfected, Infected, and Diseased.

### Sample collection

The birds were euthanized at different time points to achieve sampling for the “Uninfected,” “Infected,” and “Diseased” groups for both chicken lines. At time of euthanasia, the chickens were processed to obtain single cell suspensions of lymphocytes and mononuclear cells (WLLC) in the lungs with minor modifications. Briefly, the lungs were perfused with PBS (~ 15 ml) via oral gavage, and then the solution was collected. The procedure was repeated three times, and the collections were centrifuged at 1000 × g for 10 min at 4 °C to obtain cell pellets. The cells were resuspended in PBS and aliquoted into 2 ml vials, recentrifuged to remove the PBS, and then frozen at −80 °C. Immediately after euthanasia, ~ 5 ml of whole blood was collected by heart puncture and stored at −80 °C in heparinized tubes. Within 15 min of exsanguination, the remaining bird’s body was processed for live tissue sampling and ~ 50 mg of liver tissue was used for RNA extraction in RNA STAT-60 (Tel-Test, Inc., Friendswood, TX) and subsequently stored at − 80 °C within 30–50 min.

### Primers

Some primers have been previously described^39^ or were designed in our laboratory using previously reported procedures for primer design, primer testing, selection of internal control genes for normalization^40,41^. Briefly, gene specific primers were designed using gene bank mRNA sequences for P1 PRs [*P1A1A (ADORA1), P1A2A (ADORA2A), P1A2B (ADORA2B), P1A3 (ADORA3)*]*,* P2X PRs (*PX1- P2X7*) and P2Y PRs (*P2Y1, P2Y2, P2Y3/P2Y6, P2Y4, P2Y5, P2Y8, P2Y10, P2y12, P2Y13, P2Y14*). Primer details are provided in Table 1.

**Table 1:** Purinergic receptors primer sets used for RT-qPCR assays.

Family	Gene Name	Gene ID^1^	Accession No.^2^	Primer^3^	Sequence (5’-3’)	Fragment (bp)^4^	Source^5^
P1	ADORA1	374212	NM_204316.4	F.433	ATCAATATCGGACCGCAGAC	159	This report
				R.591	GGGTGTCACCACGCTCTTAT		
	ADORA2A	427705	XR_005854444.1	F.1574	CATTGTTGGGTTGTTTGCAG	243	This report
				R.1816	GTTCCTGCTTTGAACTGCTC		
	ADORA2B	395971	NM_205087.2	F.969	GGACTTCCGCTACACCTTCC	106	This report
				R1074	GTTGGTGACAGTCAGGTGCT		
	ADORA3	373956	NM_204151.3	F.375	GCATCCCGTTCCATTTCTGC	315	This report
				R.689	TGAGAGTCCACGCGAAGAAC		
P2X	P2X1	395190	NM_204519.2	F.312	ATACATCATAGGGTGGGTTT	359	39
				R.670	AGATTTCGCAGGTCTTCAT		
	P2X2	101749217	XM_004934420.2	F.512	CGCAGTTCACCATCCTCATC	338	39
				R.849	CTTGGCAAACCTGTAGTTGTAGC		
	P2X3	428856	NM_001397208.2	F.433	CTCGGTGTTCGTGGTGGTC	377	This report
				R.809	GCGGCTGTTGCTCGGGAT		
	P2X4	374166	NM_204291.2	F.529	CTATCACATACCCAATCCG	413	39
				R.941	TTTCAATGCCACTACTATCC		
	P2X5	395507	NM_204748.2	F.714	TCCTACCTAAAGACCTGCCACT	345	39
				R.1058	TGCCTTGCCATTCACCAT		
	P2X6	429367	XM_040685095.1	F.993	CAGCCACGCTACTCCTTCAT	207	This report
				R.1199	GGTTCCAAGGAATGCGATGC		
	P2X7	771952	XM_001235162.6	F.1300	TCCTATGATCCTCGCACCT	378	39
				R.1677	GATGATGGCTCTGTCCTCC		
P2Y	P2Y1	396275	NM_205333.2	F.326	ACCTGCCCACCGTCTACATC	348	39
				R.673	CGGCGTTCTTCTTCTTCAGC		
	P2Y2	428108	XM_025146950.2	F.38	TTTCTGCCTGCTTTACCGTCA	490	This report
				R.527	AACATGTACGTGGTGGAGGC		
	P2Y3/P2Y6	396114	NM_205195.1	F.484	AGTGCCTGCCCACCTTTGT	240	39
				R.723	CGGCCTTGTCCTTCTTCTTG		
	P2Y4	100857687	XM_004940541.4	F.13	CCGTCATTAAGCTGTCCGCA	370	This report
				R.382	CTCCTCGTTGAAGACGCACT		
	P2Y5	396118	NM_205199.3	F.556	AACAACACGGAGCAAAGAA	351	39
				R.906	CAGGGTGACAGGGTACATAG		
	P2Y8	418665	NM_001008679.2	F.669	TGGAAAGCACGGATCTGACC	569	This report
				F.1227	AGGCCATCCATAGGTCCACT		
	P2Y10	422148	XM_040669411.1	F.218	GCAACTGGACTTGCTCTGAT	653	This report
				R.870	GCTGTTGTTTTGCAGCGGTA		
	P2Y13	107049027	XM_040679499.1	F.493	GCATAGTGCTCCTCGGTCTC	662	This report
				R.1154	TGTGTCCAGTTCGGTTCTCG		
	P2Y14	101749627	NM_001317401.2	F.481	ACTCCAGCACAAACTCCTCG	524	This report
				R.1004	CTCGCTTTTAAGGCCGATGC		

^1^National Center for Biotechnology Information (NCBI) Gene ID for *Gallus gallus* (chicken).

^2^NCBI mRNA Accession No.

^3^Primer direction (F-forward; R-reverse) and hybridization position in gene.

^4^Amplicon size in base pairs.

^5^Source of primer sequence.

### RNA extraction

For WLLC samples, the cell pellet was resuspended in 500 µl RNA STAT-60 and RNA was extracted using the manufacturer’s instructions for collection of RNA. For liver samples, 50 mg of tissue sample was crushed in liquid N and the powder was suspended in 500 µl RNA STAT-60 for RNA extraction. The RNA samples were treated with DNase (Thermo Fisher Scientific), to remove any residual genomic DNA. The RNA concentrations were measured using a Nano-Drop ND-1000 spectrophotometer (Nano-Drop Technologies, Wilmington, DE, USA). The purity of RNA (A260/A280) for all samples was above 1.81 and the quality of RNA was evaluated using 2% agarose gel, and the high-quality samples were used for cDNA synthesis.

### RT-qPCR analysis

RT was performed using 2 µg DNase-treated total RNA using the High-Capacity cDNA Reverse Transcription Kit (Thermo Fisher Scientific). Twenty-microliter RT reactions were carried out according to the manufacturer’s instructions with Oligo dT20 plus random primers. The reaction mixture was incubated at 25 °C for 10 min, then 37 °C for 120 min, followed by 85 °C for 5 min. Most cDNA reactions were diluted 1:4 in ddH_2_O prior to qPCR analysis.

For qPCR, 20 µl reactions were prepared with 4 µl diluted cDNA, specific primers (0.5 μM), 10 µl 2 × Power SYBR Green Master Mix (Thermo Fisher Scientific), and ddH_2_O as previously published^40,41^. For qPCR efficiency, serial tenfold dilutions of pooled cDNA of the respective samples were used for generating standard curves, starting with approximately 500 pg of cDNA. Thermal cycling conditions were as follows: 50 °C for 2 min and 95 °C for 10 min, followed by 40 cycles at 95 °C for 15 s and 60 °C for 1 min. All RT-qPCR assays were performed using an Applied Biosystems QuantStudio 3 real-time PCR system (Thermo Fisher Scientific), and the results were analyzed using QuantStudio Design & Analysis Software v1.4.2, supplied by the manufacturer. The final genes data set were normalized with geometric mean of chicken GAPDH and 18S rRNA.

### Statistical analysis

Statistical analyses were performed using IBM SPSS Statistics version 28 software (SPSS Inc., Chicago, IL, USA). The significant differences for the plaque size assays were determined with Kruskal–Wallis tests (one-way non-parametric ANOVA), followed by multiple comparison tests. The normalized gene expression data (RT-qPCR) WLLC and liver samples were analyzed using two-way ANOVA followed by Tukey’s post-hoc tests; virus (V) and chicken breed (B) and all possible interactions (V × B) were used as fixed effects, and the relative mRNA were used as dependent variables. Statistical significance was declared at *p* < 0.05 and the mean tests associated with significant interactions (*p* < 0.05) were separated with Tukey’s tests.

### Ethical approval

All animal procedures were preapproved by the university’s Institutional Animal Care and Use Committee (IACUC) and conducted according to national regulations and ARRIVE guidelines. The animal care facilities and programs meet all the requirements of the law (89–544, 91–579, 94–276) and NIH regulations on laboratory animals, and follow the Animal Welfare Act, PL 279. The University of Illinois at Urbana-Champaign (UIUC) is accredited by the Association for Assessment and Accreditation of Laboratory Animal Care (AAALAC). Water and food were provided ad libitum.

## Results and discussion

### Characterization of v2001 in cell culture and in chickens

**Figure 2:**
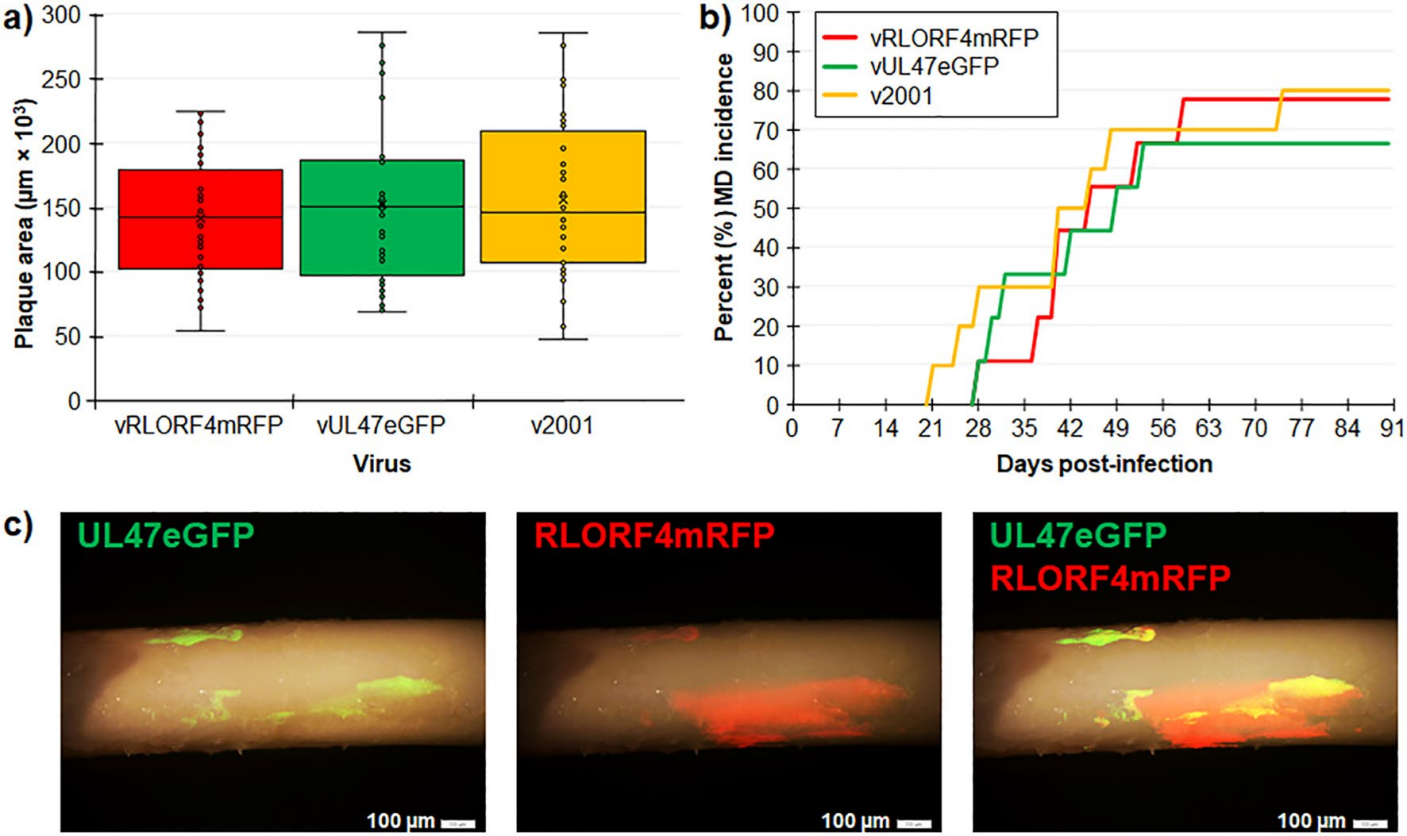
Replication of v2001 in cell culture and in chickens. (**a**) Plaque areas were measured in CECs infected with vRLORF4mRFP, vUL47eGFP, and v2001. There were no significant differences between parental viruses using ANOVA. (**b**) Total MD incidence was determined for each group. There were no significant differences in the total number of chickens developing MD in experimentally infected chickens using Fisher’s exact tests. (**c**) Feathers were plucked from v2001-infected chickens at 35 days pi and expression of RLORF4mRFP and UL47eGFP were visualized using direct fluorescent stereomicroscopy.

We previously reported that fusing fluorescent proteins to the C-terminus of the late gene pUL47 (vUL47eGFP) allows the visualization of infected cells and does not affect replication in cell culture and in vivo^30^. Similarly, we found that fusing mRFP to the C-terminus of the early RLORF4mRFP (vRLORF4mRFP) did not affect replication in cell culture and in chickens^28^. For another study, we generated a dually fluorescent virus termed v2001 by inserting eGFP at the C-terminus of the pUL47 in the previously described rRLORF4mRFP using two step Red-mediated recombination. Plaque size assays showed there were no significant differences between vRLORF4mRFP, vUL47eGFP, and the newly generated v2001 (Fig. 2a).

Next, the ability of v2001 to induce MD in chickens and transmit to contact chickens (natural infection) was tested. There was no difference between all three viruses in inducing MD in experimentally infected chickens with vRLORF4mRFP, vUL47eGFP, and v2001 inducing MD in 78, 67, and 80% of WL chickens (Fig. 2b). Additionally, all five contact chickens housed with experimentally infected chickens were positive for virus in the feathers (data not known). Importantly, both UL47eGFP and RLORF4mRFP expression could be observed in feathers of v2001-infected contact chickens (Fig. 2c). These results show that v2001 retained it virulence and transmissibility in chickens.

### The PR responses during MDV replication and MD pathogenesis

In contrast to mammals, avian macrophages are not present at the external surface of the lung’s airway epithelia in large numbers^42–44^. However, upon exposure to the infection, macrophages and other leukocytes are recruited to the surface, activating a cellular immune response in the avian respiratory tract^44,45^. During natural MDV infection, the recruitment and infection of macrophages, dendritic cells, and B and T lymphocytes is an important step in the replication of MDV and disease progression^1^; therefore, we targeted the lung’s airway for sampling in our study. WLLC is a technically easy and ideal collection of superficial, loosely attached cells from the lung epithelia^46,47^. On the other hand, the liver is one of the most important metabolic organs of the body, with up to 90% of fatty acid de novo synthesized in avian liver^48,49^. In poultry, the metabolism regulation processes, especially the lipid metabolism is similar to mammalian species with some exceptions^49–51^.

The relative expressions of three subtypes of PRs were measured in two chicken lines (Susceptible vs Resistant) in response to MDV infection and disease induction compared to uninfected controls. Based on the infection, physical condition, and internal pathology of the naturally infected susceptible (PC) and resistant (WL) birds were assigned into two different categories: Infected (MDV positive only), and Diseased (gross tumors). Uninfected birds were used as controls.

### P1 PR responses during MDV replication and MD pathogenesis

**Figure 3:**
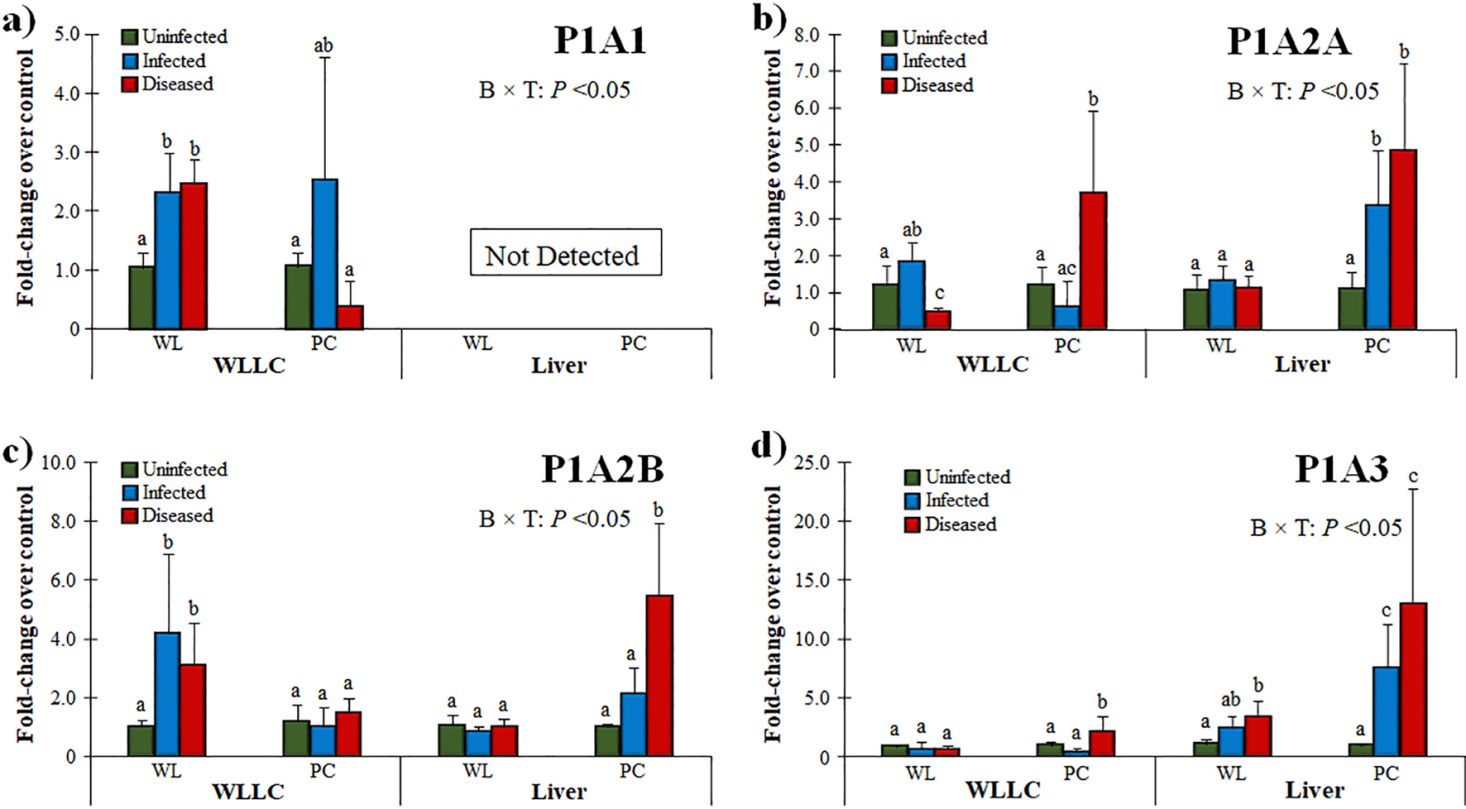
Relative mRNA expression of P1 PRs in the MD-resistant (WL) and -susceptible (PC) chicken lines. PR expression was measured in WLLC and liver samples using RT-qPCR and fold-changes compared to uninfected controls is shown for P1A1 (**a**), P1A2A (**b**), P1A2B (**c**), and P1A3 (d). Data presented as mean ± standard deviation. Statistical significance was calculated with 2-way ANOVA [Chicken line (B) × Infection (T)]. Superscripts a-c indicates significant differences (*P* < 0.05) between different groups in specific tissues (WLLCS or Liver) of the two chicken lines.

P1A1 (Adenosine A1 Receptor: ADORA1) is widely expressed in tissues, and its role has been reported to be anti-inflammatory, anti-diuretic, and involved in tissue protection, particularly the lungs and kidneys^52^. It has also been studied during Epstein-Barr herpesvirus (EBV) infection. Du et al*.*^53^ showed that cordycepin (3-deoxyadenosine), a derivative of adenosine that has anti-proliferative, anti-inflammatory, and pro-apoptotic effects, induced EBV reactivation in EBV-transformed cells. However, Ryu et al*.*^54^ showed that cordycepin suppressed EBV replication. In WLLC, the relative mRNA of P1A1 expression increased in WL for both Infected and Diseased groups compared to uninfected controls (*P* < *0.05*), while there was no difference in both PC groups (Fig. 3a). No P1A1 mRNA was detected in the liver for all groups suggesting tissue specific expression of this PR.

The higher expression in the WLLC sample of the infected birds could be associated with tissue protection (Fig. 3a). Choi and colleagues recently demonstrated that adenosine partially requires ADORA1 signaling to upregulate BZLF1, a key regulator of EBV lytic replication. Furthermore, they also confirmed that at BZLF1 upregulation by adenosine is effective in suppressing or delaying EBV-associated gastric carcinoma development^55^. In relation to the data reported here, the higher expression of ADORA1 in WLLC of Infected WL chickens (Fig. 3a) suggests they may have a more robust anti-inflammatory reaction that could be involved in their being more resistant to MD development. Further experiments are warranted to better understand the role ADORA1 plays during MDV infection and viral pathogenesis.

The expression of P1A2A significantly increased in both WLLC and liver samples from the PC Diseased group (Fig. 3b). In WLLC samples, P1A2B expression was increased in WL chickens in response to MDV infection and induction of disease, while no change was observed in PC chickens (Fig. 3c). P1A2B (Adenosine A2B Receptor: ADORA2B), along with P2Y2, have been reported to regulate mucociliary clearance, which is a dominant component of pulmonary host defenses^56^. Higher expression of these receptors in WLLC of Infected WL suggests it may play a unique role in the lungs of WL chickens. However, PC chickens had significantly higher P1A2B expression in the liver in the Diseased group. Interestingly, P1A2B signaling has been reported to contribute to immunosuppression in tumors^17^, implicating its potential role during MD progression in the susceptible PC chicken line. The higher hepatic expression of P1A2B in PC could be associated with a unique role for hepatic cells such as ADORA2B singling as anti-inflammatory and liver protection during stress and disease condition reported by different studies^57,58^.

P1A3 (Adenosine A3 Receptor: ADORA3) has been demonstrated as a mediator of anti-inflammatory, -cancer. and -ischemic protective effects. P1A3 is overexpressed in cancer and inflammatory cells, while low expression is found in normal cells^59^. In cancer cells, the activation of the P1A3A corrects an imbalance in the downstream Wnt signaling pathway^60,61^. Administration of an A3 agonist to activate its cell surface receptor inhibits the formation of cAMP and indirectly decreases phosphorylation (and therefore decreased inactivation) of the serine/threonine kinase GSK-3β. The resulting increased phosphorylation of β-catenin results in it being removed from the cytoplasm by ubiquitination; therefore, preventing its nuclear import and thus resulting inhibition of cellular proliferation that results in cell growth inhibition. With respect to cancer, nuclear factor κB (NF-κB) is a potent anti-apoptotic agent in malignant cells and its activation is strongly associated with tumors^62,63^. Whereas regulation of NF-κB has been reported as central to MDV induced pathogenesis^64^, P1A3A induces specific anti-inflammatory and anticancer effects via a molecular mechanism that entails modulation of the Wnt and the NF-κB signal transduction pathways^60,65,66^. Interestingly, P1A3 was significantly increased in both Infected and Diseased liver samples in PC chickens (Fig. 3d), suggesting a role in inflammatory and pro-tumorigenic conditions development in these chickens.

MDV has been reported to induce inflammatory cytokines (response) in the lung’s epithelium^1^. During acute stages, the P1A2A and P1A3 functions are associated as anti-inflammatory and P1A2B to vascular barrier function in the lung^67^. The higher expression of these receptors in WLLC samples (Fig. 3) in the Diseased groups for both chicken lines suggests an anti-inflammatory response to higher stress. The higher expression of P1A2A and P1A3 in the lungs of Diseased birds may help to actively regulate the active transport across the epithelial during disease stress. Similarly, the higher hepatic expression of these receptors in the Infected and Diseased MD-susceptible (PC) birds only (Fig. 3b,d), indicates a role in MD pathogenesis and in tissue protection as previously been reported in acute hepatic inflammation that could impact fibrosis progression in the liver^68–71^. Overall, the differential expression of P1 PRs in MD-susceptible and -resistant chickens and tissue specificity suggests these receptors may be involved in the complex interactions during infection and disease development during MDV infection.

### P2X PR responses during MDV replication and MD pathogenesis

**Figure 4:**
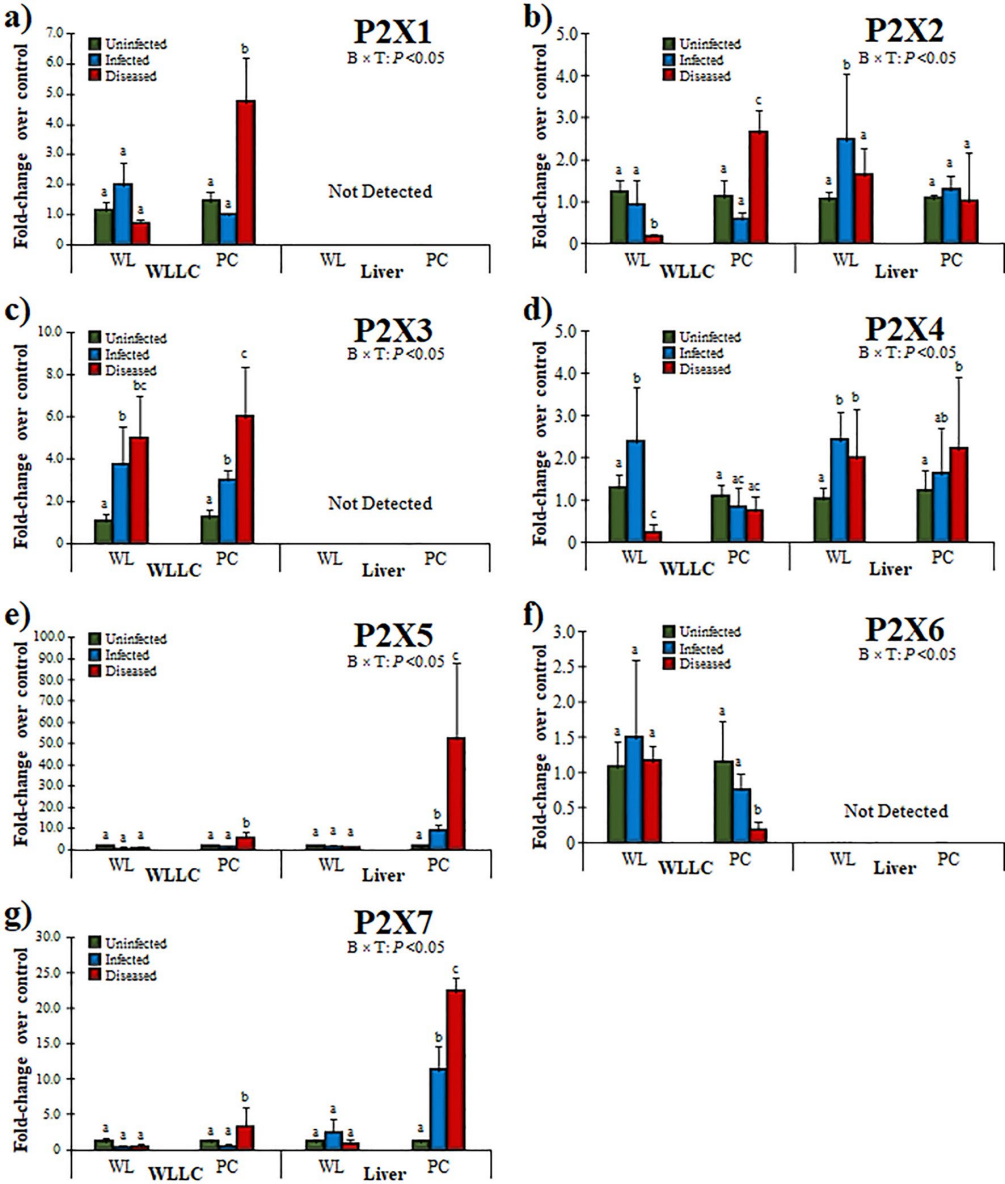
Relative mRNA expression of P2X PRs in the MD-resistant (WL) and -susceptible (PC) chicken lines. PR expression was measured in WLLC and liver samples using RT-qPCR and fold-changes compared to uninfected controls is shown for P2X1 (**a**), P2X2 (**b**), P2X3 (**c**), P2X4 (**d**), P2X5 (**e**), P2X6 (**f**), and P2X7 (**g**). Data and statistics are presented as in Fig. 3.

Overall, the expression of P2X1, P2X2, P2X3, P2X5, and P2X7 increased in WLLC samples of the Diseased PC chickens (Fig. 4). Interestingly, P2X4 was increased in Infected WL only, but was significantly decreased in Diseased birds (Fig. 4d). The expression of P2X1, P2X2, P2X5, P2X7 were significantly increased in Diseased PC birds, whereas P2X3 was increased during MDV infection, irrespective of disease condition or chicken line (Fig. 4c). In contrast, P2X6 expression decreased in Diseased PC birds, while no change was observed in WL compared to control (Fig. 4f).

Among P2X receptors, P2X2 had differential expression in the lungs of Diseased birds between the chicken lines, with WL chickens having decreased P2X2 expression, while PC birds had increased expression (Fig. 4b). WL chickens also had decreased P2X4 expression in WLLC (Fig. 4d). Interestingly, P2X5 and P2X7 were highly increased in Infected PC chickens that was further increased in the Diseased groups (Fig. 4e,g). No transcripts were detected for P2X1, P2X3, and P2X6 in the liver (Fig. 4a,c,f).

The current studies reveal higher expression of P2X PRs in Diseased MD-susceptible PC chickens (Fig. 4). Similar increases in mRNA has been reported in the EBV-infected B cells by Lee et al.^11^, that was also confirmed using western blot. There is a substantial body of evidence accumulating supporting the involvement of P2X1, P2X4 and P2X7 in host defense against infection^72^. Most immune cells, including monocytes, macrophages, dendritic, neutrophils, and T cells express P2X1, P2X4, and P2X7 during anti-pathogenic signaling^72^, suggesting an important role for these receptor subtypes in these cells. Like P2X1, the receptor can form homo- and heteromeric (*e.g*., P2X2/P2X3) ion channels for different purposes, depending on the cell type they are expressed on and the intended response^73^. P2X3 expression was absent in the liver of chickens, but abundantly expressed in WLLC (Fig. 4c), indicating an importance in the airways and lungs where ATP acts as a trigger of the cough reflex via stimulation of P2X3 and P2X2/3 receptors^74–76^. Moreover, a crucial role of P2X PRs containing P2X2 and P2X3 subunits has been reported to mediate response to hypoxia^77^. In addition, the upper and lower respiratory tract has different P2X3 distributions, such as nodose fibers expressing P2X2 and P2X3 subunits (P2X2/P2X3heterotrimers), whereas neural crest derived afferents express P2X3 and respond as P2X3 homotrimers^78–80^. The increased expression of P2X3 and P2X2 suggests MDV infection activates this response in the lungs.

Alveolar macrophages have been reported to express P2X4^81,82^. These cells phagocytize and kill microorganisms, release cytokines, and present peptides to T cells, thus triggering both cellular and humoral immune responses^83^. It has been demonstrated that P2X4-signalling promotes innate immunity in the immunopathologic response in lungs^84^. The increase in P2X4 expression in WL chickens infected with MDV that was decreased in Diseased birds (Fig. 4d) could be linked to its role in protecting against infections, inflammation, and organ injury^82^.

P2X5 is a basic requirement for the ATP-mediated inflammasome activation and IL-1β production by inflammatory stimuli^85^. The receptor has been reported as protective immune regulators during infection, mounting proper innate immune responses by regulating inflammasome activation and IL-1β production^86^. Chen et al*.*^16^ recently demonstrated that replication of HCMV was inhibited by P2X5. According to Lee et al*.*^11^, most P2 receptors are expressed on primary human B cells, while EBV-transformed cells dominantly express P2X5. In contrast to HCMV^16^, and similar to EBV^11^, the higher expression of P2X5 in MDV-Infected and Diseased PC chickens indicates a role for P2X5 in MD progression (Fig. 4e). Further research at different stages of MDV infection is warranted to delineate the role of P2X5 in the overall MD pathogenesis.

P2X7 is expressed by all tissues including different respiratory cells such as type I alveolar epithelium, pulmonary endothelial, and resident immune cells implicating it in respiratory infections. In response to pathogens, P2X7 induces alveolar macrophage activation, secretion of IL-1β and IL-1α, and neutrophil recruitment^87–89^. In addition, the receptor’s role has been linked to apoptosis by activating the inflammasome, caspases, and phospholipases^90^. The receptors also modulate intracellular signaling pathways, such as PI3K/AKT/mTOR, myeloid differentiation factor 88 (MyD88)/NF-κB, as well as the activation of mitogen-activated protein kinase (MAPK) pathway proteins (MEK, ERK 1/2)^91–93^. In the current study, the higher expression of P2X7 in MDV-infected PC chickens in both WLLC and liver (Fig. 4g) suggests an involvement in disease progression in these chickens.

### P2Y PR responses during MDV replication and MD pathogenesis

**Figure 5:**
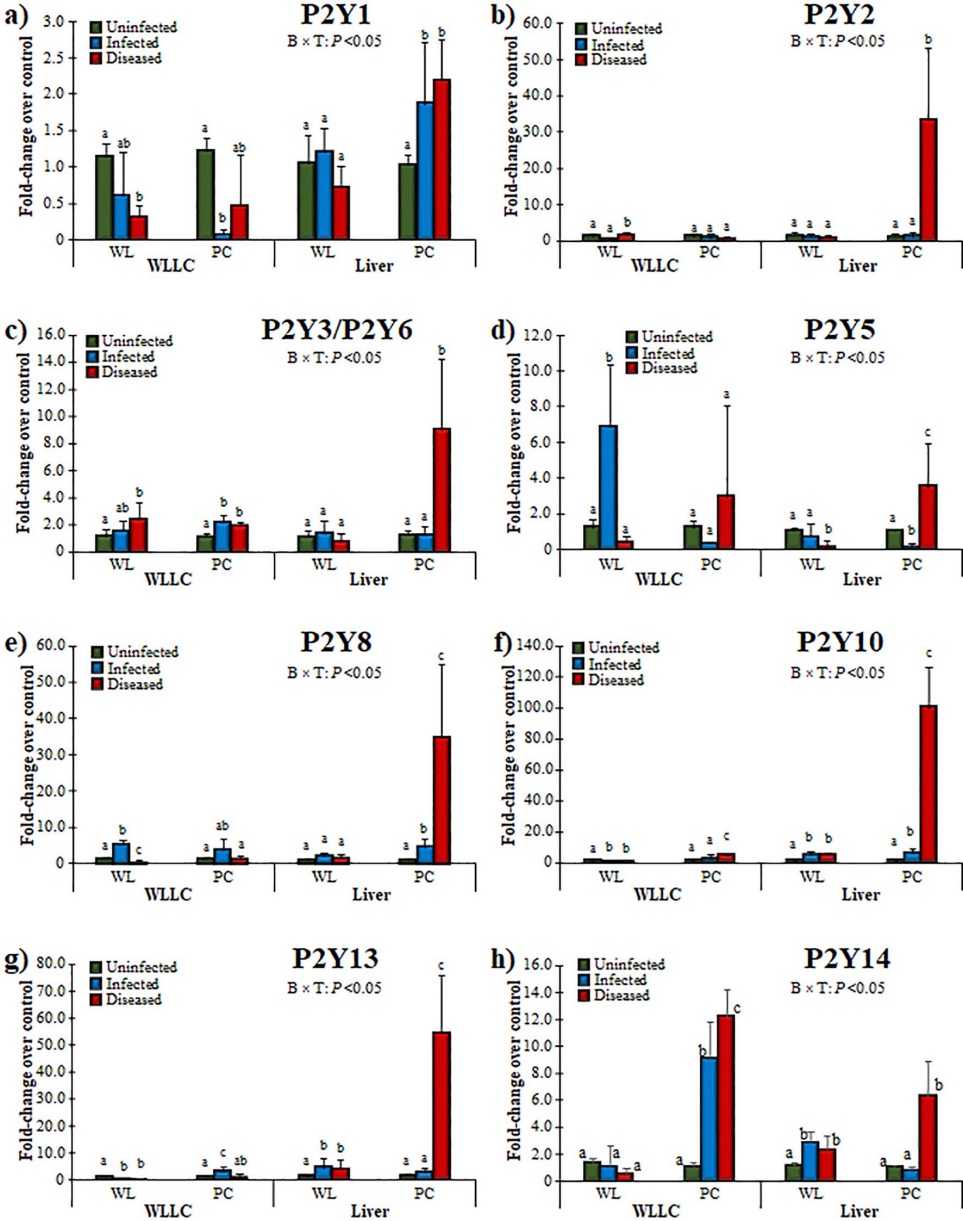
Relative mRNA expression of P2Y PRs in the MD-resistant (WL) and -susceptible (PC) chicken lines. PR expression was measured in WLLC and liver samples using RT-qPCR and fold-changes compared to uninfected controls is shown for P2Y1 (**a**), P2Y2 (**b**), P2Y3/P2Y6 (**c**), P2Y5 (**d**), P2Y8 (**e**), P2Y10 (**f**), P2Y13 (**g**), and P2Y14 (**h**). Data and statistics are presented as in Fig. 3.

In general, MDV Infected and Diseased birds had significantly affected expression of P2Y receptors (Fig. 5); however, no expression was observed for P2Y12 (not shown). The expression of P2Y1 decreased with infection in WLLC but had higher hepatic expression in MDV-Infected and Diseased PC birds (Fig. 5a). P2Y1 is widely distributed in tissues^94^ and has been reported in both innate and adaptative immune responses by inducing endothelial cell activation and leukocyte rolling^95,96^. The lower expression of P2Y1 in WLLC in both chicken lines and higher expression in Infected and Diseased PC livers implicates a tissue specific expression pattern and a role in viral replication and disease progression. Endothelial cells are an essential component of the lungs^97^ and have been reported to express P2Y1^98–100^ and exerts a protective role against infection in the lungs by ameliorating protein leakage and enhancing the proinflammatory cytokine response^101^. Therefore, the decreased expression in lungs due to MDV infection suggests MDV may downregulate its expression (Fig. 5a); however, further research is needed address the role of P2Y1 in MDV infection.

P2Y2 was significantly increased in the liver of Diseased PC chickens (Fig. 5b). A functional role for the P2Y2 ligand ATP was found in different inflammatory diseases but the overall outcome depends on the situation, infection type, and host^102^. For example, it plays a protective role in the lung during pneumonia virus infection in mice^103^. In contrast, others have reported tissue damage in the airway following inflammation and acute liver injury^104–106^. Macrophages are central during initiation of infection with different viruses, including MDV^25^. Infection triggers macrophages to perform different tasks ranging from perception of danger signals, engulfment of lipids and dead cells, secretion of reactive oxygen species (ROS) and inflammatory cytokines, and pro-resolving molecules. The macrophage-derived ROS oxidizes low-density lipoprotein (LDL) to oxidized LDL (oxLDL and stimulates IL-1β and IL-8 production^107^. Interestingly, oxLDL favors nucleotide release from endothelial cells^108^. Triggering of the P2Y2 receptor by ATP secreted by endothelial cells upon stimulation with oxLDL induces expression of receptors for advanced glycation end-products and adhesion molecules^109^. Furthermore, the release of ROS and ATP/UDP from endothelial cells upon exposure to oxLDL induce autocrine P2Y1-mediated upregulation of ICAM-1 and VCAM-1 with subsequent stimulation of leukocyte adhesion^110^. MD-susceptible and resistant chickens have significant differences in the metabolism of lipoprotein; LDL lipoprotein fraction increases with growth in the susceptible line^111^. Moreover, MD increases production of ROS leading to oxidative stress in the susceptible chickens^112,113^. Whereas this imbalance in the ambient redox level promotes oxidation of native LDL (oxLDL), thus the higher hepatic expression of P2Y1 and P2Y2 (Fig. 5a,b) in MD-susceptible Diseased birds could be linked with expected oxidation of LDL in response to stress levels.

The avian P2Y3 has been reported as an avian homologue of mammalian P2Y6^51^. For mammals, P2Y6 has been reported to contribute to airway inflammation following the induction of the allergic response in mice^114^. The antiviral role of P2Y6 has been demonstrated in different studies under different viral infections. For example, inactivated avian influenza virus-H5N1 increases IL-6 and CXCL8 mRNA by a mechanism that involves activation of P2Y6^115^. In addition, vesicular stomatitis virus **(**VSV)-induced cell death and virus replication were both enhanced significantly by knocking down or out P2Y6 in different cells^116^. In WLLC, both chicken lines showed higher expression of P2X3/P2Y6 in response to Diseased conditions (Fig. 5c), whereas, higher hepatic expression of P2Y3/P2Y6 was observed in Diseased PC birds only (Fig. 5c). These results warrant further research to understand this receptor’s potential role during the innate immune response during natural MDV infection.

P2Y5 is a G protein-coupled receptor (GPCR) that binds and is activated by lysophosphatidic acid (LPA) and by farnesyl pyrophosphate (FPP)^117^. LPA levels have been reported to increase during pathological conditions^118^, whereas LPA has been reported to regulate the Gα12/13-Rho/ROCK pathway via LPAR4/P2Y9 and LPAR6/P2Y5^119^. Former studies by Richerioux and colleagues^120^ reported that Rho-ROCK pathways regulate MDV cell-to-cell spread in cell culture. LPA has been reported to contribute to the maintenance of the epithelial integrity^121^ by regulating cellular events such renewal and migration of epithelial cells and inflammation responses^122^. LPA-mediated induction has been reported to regulate p38 MAPK, PI3K, PLC, and PKC activity and the induction of ERK1/2 phosphorylation^117^. During natural infection, WL chickens had significantly increased levels of P2Y5 in the infected lungs, while PC chickens had higher hepatic expression (Fig. 5d). These data suggest the P2Y5 response may be involved in genetic differences in response to infection in the lungs and the progression of disease based on the liver response.

The expression of P2Y8 increased (4.94 ± 1.49-fold-change) in WLLC of WL with MDV infection but was decreased (0.46 ± 0.32-fold change) in Diseased chickens (Fig. 5e). However, P2Y8 expression was significantly increased in MDV-Infected and Diseased PC chickens, while it was unchanged in WL birds. Higher expression of P2Y8 has been reported in lymphocytes, while lower expression has been shown in the lungs and other visceral organs^123^. The receptor has been shown to have oncogenic potential^124^ and fusion with other proteins like cytokine receptor-like factor 2 (CRLF2) increases its complexity in different diseases^124–126^. Similar responses were observed for P2Y5 and P2Y8 where their expression was increased in WLLC of the MD-resistant WL chickens, while being increased in the livers of the MD-susceptible PC chickens (Fig. 5d,e). Considering the potential role of P2Y8 in oncogenesis^124^, it is tempting to speculate it may play an important role in oncogenesis in the PC chicken line. These data suggest an important role in MD; however, further studies in other tissues at early exposure will help to reveal the potential role of P2Y5 and P2Y8 in MDV infection and tumorigenesis.

No expression of P2Y12 was detected in our samples, while the relative higher expression of P2Y10, P2Y13 and P2Y14 were observed in Infected PC chickens, and were further increased in the Diseased group (Fig. 5f–h). However, MDV-infected WL showed significantly lower expression of P2Y10 and P2Y13. In summary the expression of P2Y10, P2Y13 and P2Y14 expression were affected by infection and diseased PC (susceptible) chickens.

P2Y10 is another GPCR expressed by B and T cells, monocytes, dendritic cells, and granulocytes^127,128^. The putative ligands for P2Y10 are still debatable but based on different studies, the receptor has been shown to be regulated by nucleotides, LPA, sphingosine-1-phosphate (S1P) and lysophosphatidylserine (LysoPS)^129–132^. Gurusamy and colleagues demonstrated that in response to auto/paracrine acting mediators such as LysoPS and ATP, P2Y10 facilitates activation of RhoA in CD4 + T cells, thus mediating chemokine-induced migration and finally, T cell-mediated diseases^132^. Only the MD-susceptible PC chickens had increased expression of P2Y10 in both WLLC and livers (Fig. 5f) indicating a potential role in disease progression and MD susceptibility.

P2Y13 is one of the most important PRs expressed in lungs^133^ and has been reported as a potential antiviral target^19^. The receptor has been reported to improve recurrence-free survival in hepatocellular carcinoma patients^134^. Zhang et al*.*^19^ also showed that P2Y13 expression restricted the replication of both DNA (HSV-1) and RNA (NDV and VSV) viruses via JAK–STAT signaling^19^. In the current study, the higher expression (3.13 ± 1.17 fold-change) of P2Y13 during earlier infection with MDV in PC chickens in WLLC and increased expression in the liver.

of MDV-infected PC chickens (Fig. 5g) highlights the potential importance of P2Y13 as an antiviral or antitumor target as mentioned before^134^ and suggests P2Y13 may play a significant role in MD progression in different chicken lines.

The current study also revealed higher expression of P2Y14 in MDV-infected PC chickens (Fig. 5h), which indicates a potential role in pathogenesis as has been reported in other diseases^135^. This receptor is an alternative therapeutic target based on its role in many complex physiological processes like inflammation, diabetes, and immune processes^135^. Therefore, several specific potent antagonists have been developed in recent years^135^. The gene is conserved among vertebrates such as chimpanzees, rhesus monkeys, dogs, cows, chickens, and frogs. The receptor is predominately present in immune cells, and ubiquitously present in most tissues such as placenta, spleen, bone marrow, thymus, stomach, intestine, adipose tissue, and brain. Most interesting, recent data demonstrated its role in mobility and recruitment of macrophages^135^. P2Y14 can be activated by nucleotide sugar conjugates such as uridine diphosphate (UDP)-glucose (UDPG), and UDP-glucose sugars (UDPG-sugars) are potent regulators of P2Y14 to initiate subsequent signal transduction pathways via Gi/o coupled protein^136^. UDPG has been reported to promote neutrophil and macrophage recruitment in the lung^137^. Interestingly, it has been documented that MDV increases the number of macrophages in the lungs of the infected chickens^138^. IFN-γ has been shown to be associated with immunity against MDV^139^. Moreover, IFN-γ treatment stimulates glycogen synthesis in macrophages that is channeled through glycogenolysis to generate G6P and further to NADPH, ensuring high levels of reduced glutathione for inflammatory macrophages survival^140^. This in turn, leads to increased UDPG levels and ultimately P2Y14 expression in macrophages^140^. Gilfernandez and colleagues^141^ completely abolished the replication of HSV-2 and African swine fever virus using a uridine 5'-diphosphate glucose analogues at 100 and 150 µg/ml, respectively. These all suggest a key role of P2Y14 in MD and potentially during recruitment of macrophages or other immune cells to the lungs.

## Conclusions

In the current study, we measured the PR response during MDV infection. The findings from our study suggests the genetics of the host plays a key role during MDV infection and disease progression. Selection and breeding of chickens for relative resistance and susceptibility to MD has been a major parameter for the poultry industry^7^. Thus, using two different chicken lines (MD-susceptible and MD-resistant) were used here to determine whether the PR response was different in these two chicken lines. Interestingly, differential regulation in many PR was observed between MD-resistant and –susceptible chickens. Our study revealed interesting results regarding the differential expression of PRs in response to MDV infection and severity of MD. Both tissues and chicken breed had different expression patterns of PRs. The different expression levels confirmed that some PRs expression changes with pathophysiological conditions and tissue type. In general, the PRs involved in tissue protection and disease control were increased in MD-resistant WL chickens, whereas PRs involved in disease progression were increased in MD-susceptible PC chickens. As far as we are aware, this is the first study evaluating PR responses to natural MDV infection and MD pathogenesis and suggests further studies are warranted to elucidate the PR response to MD.

## Data Availability

The authors declare that all data supporting the findings of this work are available within the paper.
